# Embigin Is Highly Expressed on CD4^+^ and CD8^+^ T Cells but Is Dispensable for Several T Cell Effector Responses

**DOI:** 10.4049/immunohorizons.2300083

**Published:** 2024-03-06

**Authors:** Haoran Yang, Naoki Iwanaga, Alexis R. Katz, Andy R. Ridley, Haiyan D. Miller, Michaela J. Allen, Dereck Pociask, Jay K. Kolls

**Affiliations:** * Department of Medicine, Center for Translational Research in Infection and Inflammation, Tulane University School of Medicine, New Orleans, LA; † Department of Pediatrics, Center for Translational Research in Infection and Inflammation, Tulane University School of Medicine, New Orleans, LA; ‡ Department of Respiratory Medicine, Nagasaki University Hospital, Nagasaki, Japan; § Department of Medicine, Tulane University School of Medicine, New Orleans, LA

## Abstract

T cell immunity, including CD4^+^ and CD8^+^ T cell immunity, is critical to host immune responses to infection. Transcriptomic analyses of both CD4^+^ and CD8^+^ T cells of C57BL/6 mice show high expression the gene encoding embigin, *Emb*, which encodes a transmembrane glycoprotein. Moreover, we found that lung CD4^+^ Th17 tissue-resident memory T cells of C57BL/6 mice also express high levels of *Emb*. However, deletion of *Emb* in αβ T cells of C57BL/6 mice revealed that *Emb* is dispensable for thymic T cell development, generation of lung Th17 tissue-resident memory T cells, tissue-resident memory T cell homing to the lung, experimental autoimmune encephalitis, as well as clearance of pulmonary viral or fungal infection. Thus, based on this study, embigin appears to play a minor role if any in αβ T cell development or αβ T cell effector functions in C57BL/6 mice.

## Introduction

Cells in multicellular organisms are dispersed throughout; not all are in physical contact with one other. For cells to function, they must be able to respond to cues from their local microenvironment, distant hormones, as well as external stimuli. This intricate signaling network is facilitated by the involvement of four key types of adhesion molecules, namely cadherins, integrins, selectins, and members of the Ig superfamily (IgSF) (1). The IgSF recognizes additional IgSF members as well as integrins as their ligands. Such heterophilic interactions between endothelial cells and selectins—found on leukocytes, platelets, and additional endothelial cells–are integral for cell integrity, diapedesis, and thrombus formation (2). Furthermore, these adhesion molecules play a vital role in signal transduction, forming a dynamic interface that governs cellular responses and interactions.

Embigin (encoded by *Emb*) is a transmembrane glycoprotein within the IgSF that has two Ig domains (3–5). It has been shown to be expressed in sebocyte progenitors in the sebaceous gland and provides structural support of these cells through binding to fibronectin (6). Studies have linked embigin to pancreatic adenocarcinoma and breast cancer, with both organs containing sebaceous glands (7, 8). Additionally, it has been found to play a role in early rat prostate and mammary gland development (5), further suggesting its potential epithelial niche-interacting factor.

It has been reported that embigin assists in the transportation of nutrients by acting as a chaperone for monocarboxylate transporters (MCTs) (9). These MCTs are necessary for the bidirectional flow of monocarboxylates—such as lactate, β-hydroxybutyrate, and acetoacetate—across the plasma membrane (10). Cells that use glycolysis as a major source of energy, such as leukocytes, must remove lactic acid to prevent acidosis. Of the four members of the MCT family, MCT2 preferentially binds to its chaperone protein embigin (11). MCT2 is found in the spleen, heart, kidney, and brain and in leukocytes (12, 13). Previous studies have demonstrated that inhibition of MCT1 impairs mouse T cell proliferation in vitro, although studies focusing on MCT2 function with regard to T cell function are limited (14).

Homeostasis of the hematopoietic stem and progenitor cell (HSPC) microenvironment is based on the adhesive interactions of HSPCs with nearby cells, hormones, growth factors, and components of the extracellular matrix (ECM). These adhesive interactions influence HSPC self-renewal and proliferative potential. Embigin is one of the components that makes up the ECM in the HSPC microenvironment (15). Recent studies have revealed that embigin serves a role in the regulation and retention of HSPCs via regulation of HSPC quiescence (16). *Emb* transcripts are highly expressed in the brain, visceral yolk sac, and foregut during mouse embryogenesis (3).

Cell adhesion molecules play an important role in cell attachment, migration, and metastases. Integrins are known to bind to and activate MMP2, leading to the breakdown of the ECM; this plays a pivotal role in angiogenesis. Furthermore, integrin regulates cell attachment, spreading, and migration, playing a role in metastasis. In endothelial cells, integrin prevents apoptosis through the intrinsic apoptosis pathway (17). With integrin as its ligand, one could postulate that embigin is associated with malignancy*. Emb* has been found to be expressed in a variety of cancer cell types, including pancreatic and breast cancer. Embigin suppresses tumorigenesis in breast cancer cells while promoting pancreatic cancer progression (7, 8). Homeobox C8 binds to the *Emb* promoter and inhibits *Emb* expression, which leads to an increase in proliferation, anchorage-independent growth, and migration of breast cancer cells (8). *Emb* expression is elevated in pancreatic ductal adenocarcinoma and involved in epithelial-to-mesenchymal transition in pancreatic cancer via the TGF-β signaling pathway (7).

Data from ImmGen (18) show that *Emb* is highly expressed in both αβ and γδ T cells. Prior work from our group showed that *Emb* is also highly expressed on lung CD4^+^ tissue-resident memory T (TRM) cells (19) after immunization with the adjuvant *Escherichia coli* labile toxin A1 (LTA1) and outer membrane protein X (OmpX) from *Klebsiella pneumoniae*. Also, a recent study has demonstrated that *Emb* is highly expressed in skin-resident NK cells (20). However, the function of *Emb* in T cells remains unclear. To study this, we generated *Cd4cre* × *Emb^fl/fl^* mice and used several CD4^+^ T cell–dependent models to phenotype these mice, including lung TRM cell generation by mucosal vaccination, the lung homing property of lung TRM cells, the experimental autoimmune encephalomyelitis (EAE) model, *Pneumocystis* infection, as well as generation of CD8^+^ T cell memory after influenza infection. Despite loss of embigin protein, embigin was found to be dispensable for both generation of CD4 responses as well as generation of CD8^+^ T cell memory.

## Materials and Methods

### Mice

*Emb^fl/fl^* mice were generated by Cyagen (Santa Clara, CA). *Cd4-cre^+^* mice (B6.Cg-Tg(Cd4-cre)1Cwi/BfluJ) and B6.SJL-*Ptprc^a^ Pepc^b^*/BoyJ mice (CD45.1^+^ C57BL/6) were obtained from The Jackson Laboratory. Male and female mice (6–12 wk old) were used for studies. All mice were housed and bred at the Tulane University Department of Comparative Medicine Facility. Animals were housed in a pathogen-free environment. All experiments were performed using sex- and age-matched controls and approved by the Institutional Animal Care and Use Committee of Tulane University.

### ImmGen data analysis

*Emb* expression data were obtained through ImmGen. The CD4^+^CD8^−^CD24^int^TCRb^hi^ cells, CD4^−^CD8^+^CD24^int^TCRb^hi^ cells, and CD19^+^IgM^+^ B cells from C57BL6/6J mice were sorted for bulk RNA sequencing. The sequencing data were normalized by DESeq2 (21).

### Ag and adjuvant preparation for immunization

We have previously shown that immunization with OmpX and LTA1, a mucosal Th17 adjuvant, derived from the A1 domain of the heat-labile toxin from *Escherichia coli*, drives the production of OmpX-specific lung CD4^+^ TRM cells (19, 22). To investigate whether Emb is required in CD4^+^ lung TRM cell generation, we performed LTA1/OmpX immunization with isoflurane-anesthetized mice using the oropharyngeal aspiration–tongue pull technique and boosted with the same vaccine 3 wk later. *Emb^fl/fl^Cd4-cre*^−^ and *Emb^fl/fl^Cd4-cre^+^* mice (male, 6–10 wk old) were used for this experiment.

### Experimental *K. pneumoniae* infection

*K. pneumoniae*-396 (K1 strain) was prepared as previous reported (19). Briefly, *K. pneumoniae*-396 (K1 strain) was grown in 30 ml of tryptic soy broth (Difco) overnight at 37°C with shaking at 233 rpm. Cultures were then diluted at 1:100 and grown in the same conditions for 2.5 h to achieve early logarithmic phase. The concentration of *K. pneumoniae* was determined by measuring the OD at 600 nm. Bacteria were pelleted and washed twice in cold PBS and then resuspended in PBS to the desired concentration. Mice were infected with 1 × 10^4^ CFU intratracheally and sacrificed at 24 h postinfection. The lungs and spleens were homogenized and diluted in PBS, then plated on Luria-Bertani agar plates for CFU.

### Induction and clinical evaluation of EAE

For the induction of EAE, *Emb^fl/fl^Cd4-cre^−^* and *Emb^fl/fl^Cd4-cre^+^* mice (female, 9–13 wk old) were treated with myelin oligodendrocyte glycoprotein (MOG)_35–55_/CFA emulsion pertussis toxin (no. EK-2110, Hooke Laboratories) following the manufacturer’s instructions. Mice were examined daily for signs of EAE and scored as follows: 0, no disease; 1, tail paralysis; 2, hindlimb weakness; 3, hindlimb paralysis; 4, hindlimb plus forelimb paralysis; 5, moribund or dead.

### Single-cell suspension preparation

Single-cell suspensions of spinal cords were prepared as previously reported (23). Spinal cords were isolated and placed in ice-cold RPMI 1640 medium containing 27% Percoll, and pressed through a 70-μm cell strainer (Fisher Scientific). The resulting cell suspension was brought to a volume of 50 ml with 27% Percoll, mixed, and centrifuged at 300 × *g* for 15 min. The pellet was kept on ice, while the myelin layer and the supernatant were transferred to a new 50-ml tube, homogenized by shaking, and centrifuged again at 300 × *g* for 15 min. The pellets were then combined and washed three times in RPMI 1640 medium at 4°C. Single-cell suspensions of spleens were prepared by being pressed through a 70-μm cell strainer (Fisher Scientific) and centrifuged at 300 × g for 5 min. ACK lysis buffer (Life Technologies) was added to the cell pellet for 4 min and washed then resuspended in complete IMDM medium.

The lungs were minced manually with dissection scissors and digested in 4 ml IMDM (Life Technologies) with 2 mg/ml collagenase (Sigma-Aldrich) and 80 U/ml DNase1 (Sigma-Aldrich) at 37°C for 1 h. Digested tissue was strained through a 70 μm cell strainer (Fisher). ACK (ammonium-chloride-potassium) lysis buffer (Life Technologies) was added to the cell pellet for 4 min and then washed and resuspended in complete IMDM medium.

### *Pneumocystis* infection and quantification

*Emb^fl/fl^Cd4-cre^−^* and *Emb^fl/fl^Cd4-cre^+^* mice (female, 6–10 wk old) were infected with *Pneumocystis murina* (2 × 10^5^ cysts) via oral pharyngeal administration. Mice were euthanized 2 and 6 wk later by reverse transcription–quantitative PCR (RT-qPCR) to assess fungal burden as previously described (24, 25).

### IFN-γ ELISPOT assays

ELISPOT assays were conducted in Millipore pore plates to evaluate the frequency of background and Ag-stimulated spot forming units using an ELISPOT Flex: mouse IFN-γ (ALP) kit (no. 3321-2A, Mabtech). Lung cells (10^5^ per well) were stimulated with 2 μg/ml *Pneumocystis* in triplicate.

### Intracellular cytokine staining and flow cytometry

Single-cell suspensions from mouse lungs and spleens were stimulated for 5 h with PMA (50 ng/ml; Sigma-Aldrich) and ionomycin (750 ng/ml; Sigma-Aldrich) and GolgiStop (1 mg/ml; BD Biosciences). Cells were then stained with Abs specific for surface markers, followed by permeabilization/fixation with Cytofix/Cytoperm (BD Biosciences) and stained with Abs against intracellular molecules. The cells were analyzed using a Cytek Aurora spectral flow cytometer. The influenza nuclear protein MHC class I (H-2D^b^/ASNENMETM) tetramer (conjugated to PE) was obtained from the National Institutes of Health Tetramer Core Facility (Emory University, Atlanta, GA). Staining was carried out at room temperature for 1 h in conjunction with other surface staining.

Abs used for blocking and staining are as follows: rat anti-mouse CD16/CD32 Fc Block (clone 2.4G2, BD Biosciences), PE-Cy7 rat anti-mouse CD4 (clone RM4-5, BD Biosciences), allophycocyanin rat anti-mouse CD3e (clone 17A2, BioLegend), PE-Cy5 hamster anti-mouse TCRβ (clone H57-597, BD Biosciences), FITC rat anti-mouse IL-17A (clone TC11-18H10.1, BioLegend), Brilliant Violet 421 rat anti-mouse IFN-γ (clone XMG1.2, BioLegend), eFluor 450 rat anti-mouse CD19 (clone 1D3, Invitrogen), PE rat anti-mouse embigin (clone G7.43.1, eBioscience), allophycocyanin rat anti-mouse CD8a (clone 53-6.7, BD Biosciences), and FITC mouse anti-mouse CD45.2 (clone 104, BioLegend). Regulatory T cell (Treg) staining was conducted using a True-Nuclear mouse Treg flow kit (Foxp3 Alexa Fluor 488/CD4 allophycocyanin/CD25 PE) kit (no. 320029, BioLegend).

### Adoptive transfer of CD4^+^ T cells

*Emb^fl/fl^Cd4-cre^−^* and *Emb^fl/fl^Cd4-cre^+^* mice (CD45.2^+^) were immunized as mentioned. One week after second immunization, the lungs were removed and digested into the single-cell suspensions, and CD4*^+^* T cells were enriched by using a CD4 positive selection kit (no. 130-117-043, Miltenyi Biotec). Wild-type (WT) C57BL/6 mice (CD45.1^+^) were transferred with the enriched 5 × 10^5^ immunized lung CD4^+^ T cells via retro-orbital vein 1 d after inoculation with 1 μg of OmpX and 10 μg of LTA1 intratracheally.

### Influenza infection

Influenza A/PR/8/34 H1N1 was propagated in chicken eggs as previously described (26). *Emb^fl/fl^Cd4-cre^−^* and *Emb^fl/fl^Cd4-cre^+^* mice (male, 8–12 wk old) were infected with 30 plaque-forming units of influenza virus in 50 μl of sterile PBS intratracheally. Following infection, mice were monitored daily for weight loss for 7 d and survival. At 60 d postinfection, lung cells were harvested for flow cytometry.

### Illumina mRNA library preparation

Prior to Illumina mRNA library construction, DNase-related total RNA was quantitated using the Qubit RNA HS (high-sensitivity) assay kit (no. Q32855, Thermo Fisher Scientific,). RNA quality (RNA integrity number) was determined on an Agilent TapeStation 4150 using Agilent RNA ScreenTape (no. 5067-5576, Agilent), after which 0.3 μg of each total RNA (RNA integrity number > 8) was applied to generate mRNA libraries using an Illumina TruSeq stranded mRNA sample preparation kit (no. 20020594, Illumina), following the Illumina TruSeq stranded mRNA sample preparation guide (Illumina document no. 100000004049). Final cDNA libraries containing TruSeq RNA CD indexes (no. 20019792, Illumina) were quantitated using a Qubit dsDNA HS (high-sensitivity) assay kit (no. Q32854, Thermo Fisher Scientific). The quality of the libraries was determined by running each on an Agilent TapeStation 4150 using an Agilent D1000 ScreenTape (no. 5067-5582, Agilent). Smear analysis was performed using Agilent TapeStation software (version 4.1.1) with a range of 200–600 bp to determine average size of each library. Size and concentration were then used to calculate the molarity of each library. All libraries were pooled at a final concentration of 750 pM with a spike-in of 2% PhiX Control v3 library (no. FC-110-3001, Illumina). Mixture of pooled libraries was loaded on an Illumina NextSeq P1 (300) reagent cartridge (no. 20050264, Illumina). Paired-end and dual indexing sequence, 150 × 8 × 8 × 150, was performed on NextSeq2000, yielding ∼20 million paired-end reads per sample. Fastqs generated by Illumina BaseSpace DRAGEN analysis software (version 1.2.1) were applied for further data analyses. RNA sequencing data were deposited in Gene Expression Omnibus under accession number GSE253411 (https://www.ncbi.nlm.nih.gov/geo/query/acc.cgi?acc=GSE253411).

### Statistical analysis

Statistical analysis was performed with Prism (GraphPad) software. For comparison between two groups, a Student *t* test was used. For analysis comparing three or more groups, we used one-way ANOVA with Tukey post hoc analysis. For analyses involving bacterial burdens, we performed a log transformation on the data and performed ANOVA on transformed data. A *p* value <0.05 was considered statistically significant. The *p* values are annotated as follows: *p ≤ 0.05, **p ≤ 0.01, ***p ≤ 0.001, ****p ≤ 0.0001.

## Results

### *Emb* is highly expressed on CD4^+^ T cells

**FIGURE 1.**
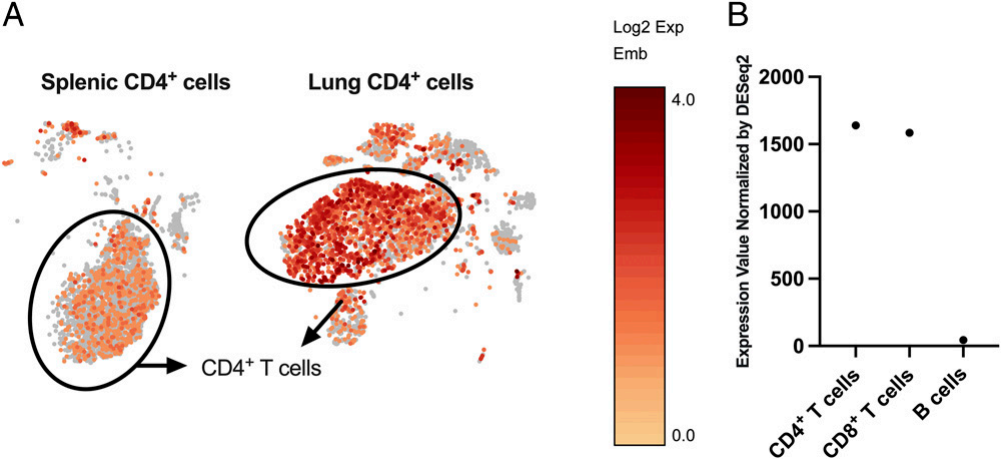
Embigin is highly expressed on CD4^+^ T cells. (**A**) Representative *t*-distributed neighborhood stochastic embedding plot of embigin expression in splenic CD4^+^ T cells (left) and lung CD4^+^ TRM cells (right) by single-cell RNA sequencing (*n* = 2 per group from one experiment). (**B**) Normalized expression level by DESeq2 of *Emb* in murine CD4^+^ T cells, CD8^+^ T cells, and B cells from the ImmGen dataset. The CD4^+^CD8*^−^*CD24^int^TCRb^hi^ cells (*n* = 2), CD4*^−^*CD8^+^CD24^int^TCRb^hi^ cells (*n* = 2), and CD19^+^IgM^+^ B cells (*n* = 1) from C57BL/6J were sorted for bulk RNA sequencing.

Previous work has shown that OmpX and LTA1 together elicit Ag-specific CD4^+^ TRM cells in the lung, which can protect the lung against *K. pneumoniae* infection (19). Single-cell RNA sequencing analysis showed that the CD4^+^ TRM cells highly expressed *Emb*. CD4^+^ T cells from naive spleen also expressed *Emb* but to a lower extent (Fig. 1A). Data from ImmGen showed that *Emb* was also highly expressed on CD4^+^ and CD8^+^ T cells. The expression level of *Emb* on B cells was low (Fig. 1B) (21). However, there is currently no report about the function of *Emb* on CD4^+^ and CD8^+^ T cells.

### *Cd4cre × Emb^fl/fl^* mice generation and validation

To study the function of *Emb* on T cells, we generated *Emb^fl/fl^* mice. The *Emb* gene is located on mouse chromosome 13. Nine exons were identified, with the ATG start codon in exon 1 and the TGA stop codon in exon 9. Exon 5 was selected as the conditional knockout (cKO) region. The KO of exon 5 resulted in a frameshift mutation. To engineer the targeting vector, homologous arms and the cKO region were generated by PCR using BAC (bacterial artificial chromosome) clone RP23-135K13 as a template. In the targeting vector, the Neo cassette was flanked by SDA (self-deletion anchor) sites. DTA (diphtheria toxin A) was used for negative selection. C57BL/6N embryonic stem cells were used for gene targeting (Supplemental Fig. 1A). After *Emb^fl/fl^* mice were generated, they were crossed to *Cd4-cre^+^* mice to conditionally knockout *Emb* on CD4*^+^* T cells. Loss of embigin expression in lung TRM cells in *Cd4cre* × *Emb^fl/fl^* mice was validated immunizing mice with LTA1 and OmpX as previously described (19) and staining for surface embigin by flow cytometry (Supplemental Fig. 2). We found high levels of embigin expression in *Cre^−^* mice (WT), which is substantially reduced in *Cre^+^* mice (KO). Similar results were found on the CD4^+^ and CD8^+^ T cells from spleen and thymus (Supplemental Fig. 1B).

### *Emb* is dispensable for T and B cell development

**FIGURE 2.**
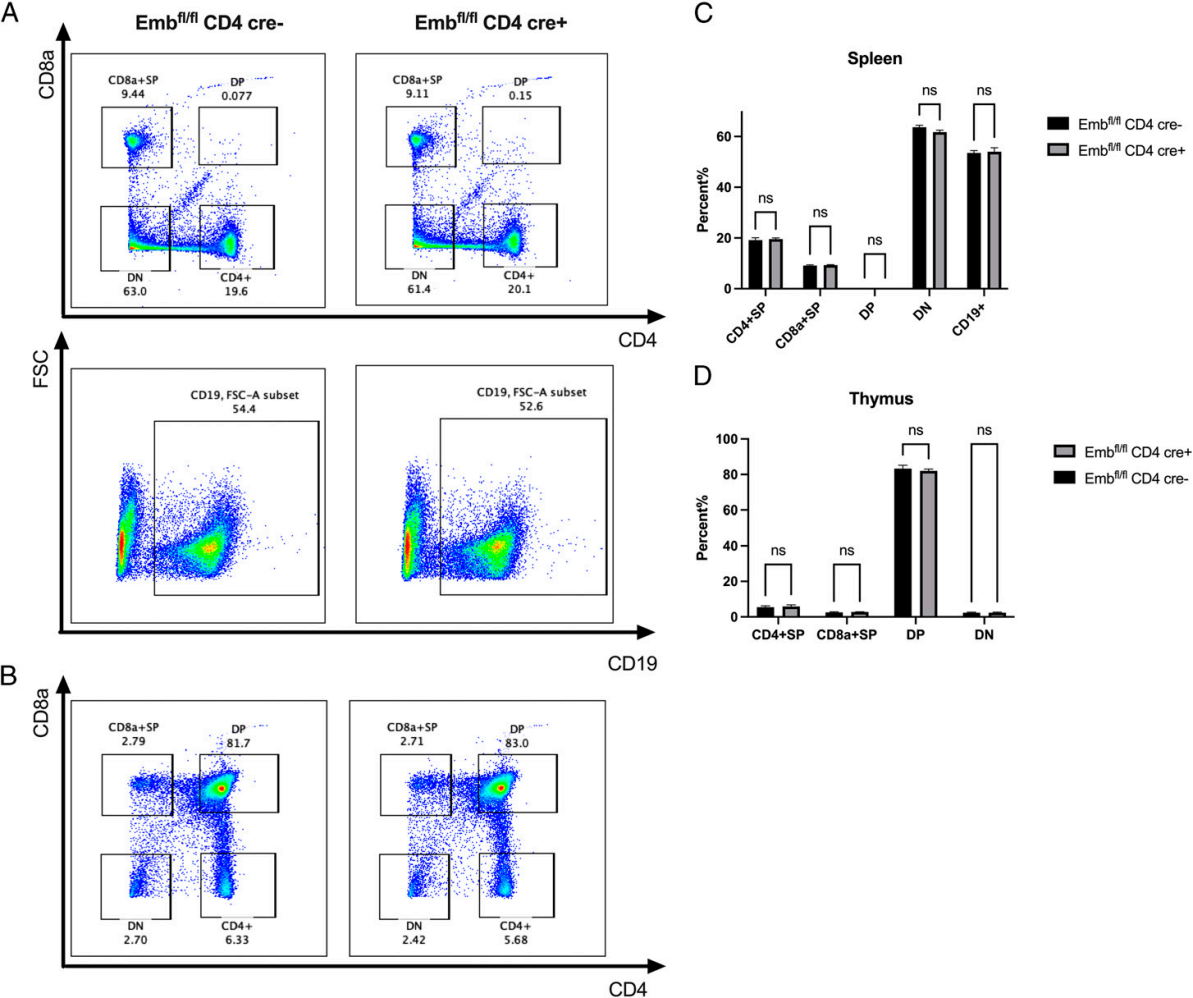
T and B cell development in *Cd4cre* × *Emb^fl/fl^* mice. (**A**) Representative dot plots of spleen CD4^+^ T cells, CD8a^+^ T cells, CD4*^−^*CD8a*^−^* T cells, CD4^+^CD8a^+^ T cells, and CD19^+^ B cells in *Emb^fl/fl^ CD4-cre^−^* (WT) and *Emb^fl/fl^ CD4-cre^+^* (KO) mice. (**B**) Representative dot plots of spleen CD4^+^ T cells, CD8a^+^ T cells, CD4*^−^*CD8a*^−^* T cells, and CD4^+^CD8a^+^ T cells in WT and KO mice. (**C** and **D**) Quantitative data of (A) and (B) (*n* = 3 per group from one experiment). Data are presented as means ± SEM. Significant differences were calculated with an unpaired Student *t* test.

To study the function of *Emb* on T and B cell development, we harvested the spleen and thymus from 4-wk-old WT and *Emb*-deficient mice. In terms of the number of CD4^+^ T cells, CD8a^+^ T cells, CD4*^−^*CD8a*^−^* T cells, CD4^+^CD8a^+^ T cells and CD19^+^ B cells, there were no significant differences between WT and KO mice in the spleen (Fig. 2A, 2C) and thymus (Fig. 2B, 2D). These results suggest that *Emb* deficiency does not affect T and B cell development.

### *Emb* is dispensable for LTA1-induced CD4^+^ TRM cells and vaccine efficacy

**FIGURE 3.**
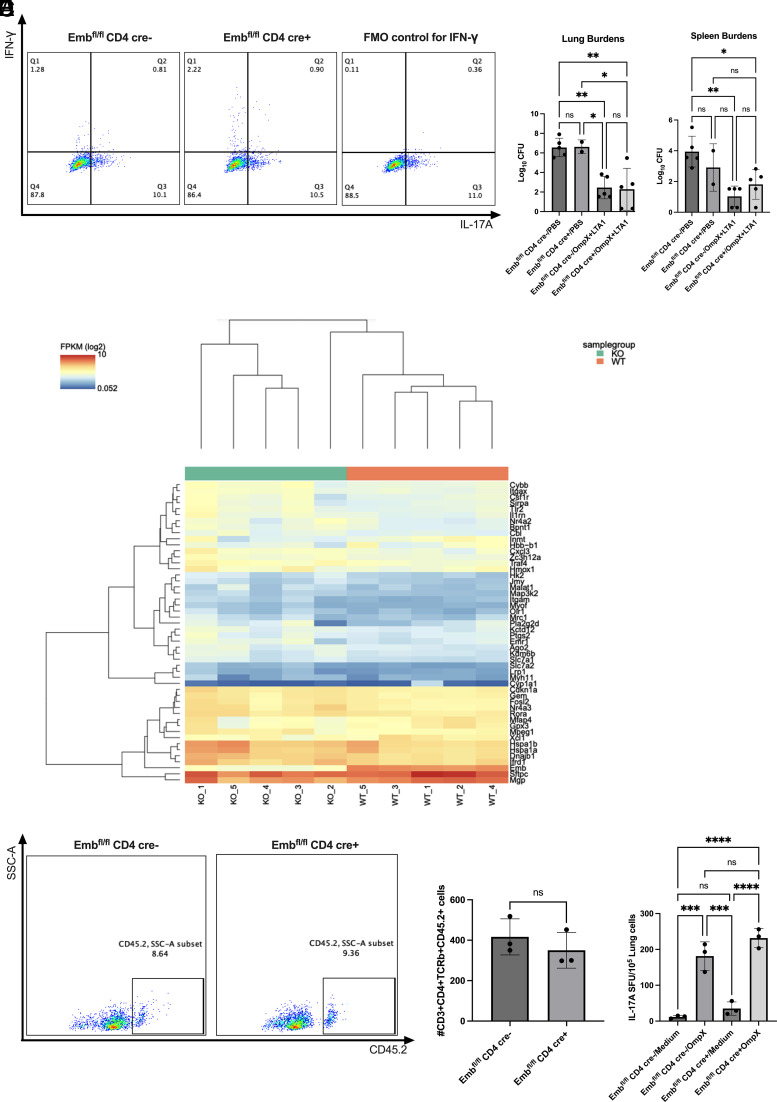
Embigin is dispensable for LTA1-induced CD4^+^ TRM cells and vaccine efficacy. (**A**) WT and KO mice were immunized twice with 1 μg of OmpX and 10 μg of LTA1 intratracheally 3 wk apart. Four weeks after the prime immunization, the mice were challenged with 10^4^ CFU of *K. pneumoniae* (K1 strain), and bacterial burden (CFU) in lungs and spleens was determined at 24 h postinfection (*n* = 2–5 per group, representative from two independent experiments). (**B**) Representative dot plots of lung IL-17a^+^ cells, IFN-γ^+^ in WT and KO mice. (**C**) CD4^+^ T cells were enriched from immunized WT and KO mice and processed for RNA sequencing. Heatmap of Cuffdiff result is shown (*n* = 5 per group, from one experiment). (**D**–**F**) Splenic CD4^+^ T cells from OmpX+LTA1–vaccinated WT and KO mice were purified on day 29 after OmpX+LTA1 vaccination. CD4^+^ T cells (5 × 10^5^) were i.v. transferred into CD45.1^+^ C57BL/6 mice (*n* = 3 per group, representative from one independent experiment). (D) Representative dot plots of CD45.2^+^CD3^+^CD4^+^TCRβ^+^ cells in CD45.1^+^ C57BL/6 mice after transfer. (E) Absolute number of CD45.2^+^CD3^+^CD4^+^TCRβ^+^ cells in CD45.1^+^ C57BL/6 mice after transfer. (F) IL-17A ELISPOT frequency to detect OmpX-specific CD4^+^ T cells from the transferred CD45.1^+^ mice. Data are presented as means ± SEM. Significant differences were calculated with one-way ANOVA followed by a Tukey multiple comparison test. *p ≤* *0.05, **p ≤* *0.01, ***p ≤* *0.001, ****p ≤* *0.0001.

It has been reported that the LTA1-induced CD4*^+^* T cells are TRM cells that highly express *Cd44* and *Cd69* (19). Because *Emb* is highly expressed in LTA1-induced CD4*^+^* TRM cells, we immunized and challenged WT and KO mice as previously described with LTA1/OmpX (19). Single cells from the mouse lung were generated and IL-17A and IFN**-**γ production levels were validated by flow cytometry. In WT mice, ∼10% of CD4^+^ T cells were IL-17A–producing cells, which was comparable in KO mice (Fig. 3A). Furthermore, after *Klebsiella* challenge, both WT and KO mice showed equivalent protection in the lung and spleen compared with the naive control (Fig. 3B). Intracellular staining for IFN**-**γ was validated on in vitro–differentiated Th1 cells because the IFN**-**γ^+^ cell number is low in the lung CD4^+^ T cells (Supplemental Fig. 3). To determine whether the *Emb*-deficient CD4^+^ T cells differ from WT CD4^+^ T cells, we enriched the lung CD4^+^ T cells from immunized WT and KO mice using magnetic beads. We performed bulk RNA sequencing on these cells and found the differentially expressed genes between WT and KO mice (Fig. 3C). Interestingly, *Tlr2* and *Il1α* were elevated in *Emb*-deficient CD4*^+^* T cells, which indicated that the Tlr2 signaling pathway might be associated with *Emb* regulation. Embigin has been reported to function as a cell adhesion molecule and plays a role in cell migration (6, 7). Also, we have previously shown that LTA1-induced CD4^+^ TRM cells can home back to the lung after adoptive transfer (19). Therefore, we tested whether *Emb* deficiency affects the homing of adoptively transferred CD4*^+^* TRM cells. CD4*^+^* T cells were enriched by magnetic beads from the lungs of immunized WT or KO mice. The CD4^+^ T cells were adoptively transferred to Cd45.1^+^ mice i.v. as previously described (19). We observed no differences between WT and KO mice in terms of the number of CD45.2^+^ cells found in the mouse lung by flow cytometry (Fig. 3D, 3E). Moreover, the frequency levels of IL-17A–producing cells by ELISPOT (Fig. 3F) were similar, suggesting that *Emb* is dispensable of lung TRM cell homing in this model (Fig. 3E).

### *Emb* is dispensable for EAE pathogenesis

**FIGURE 4.**
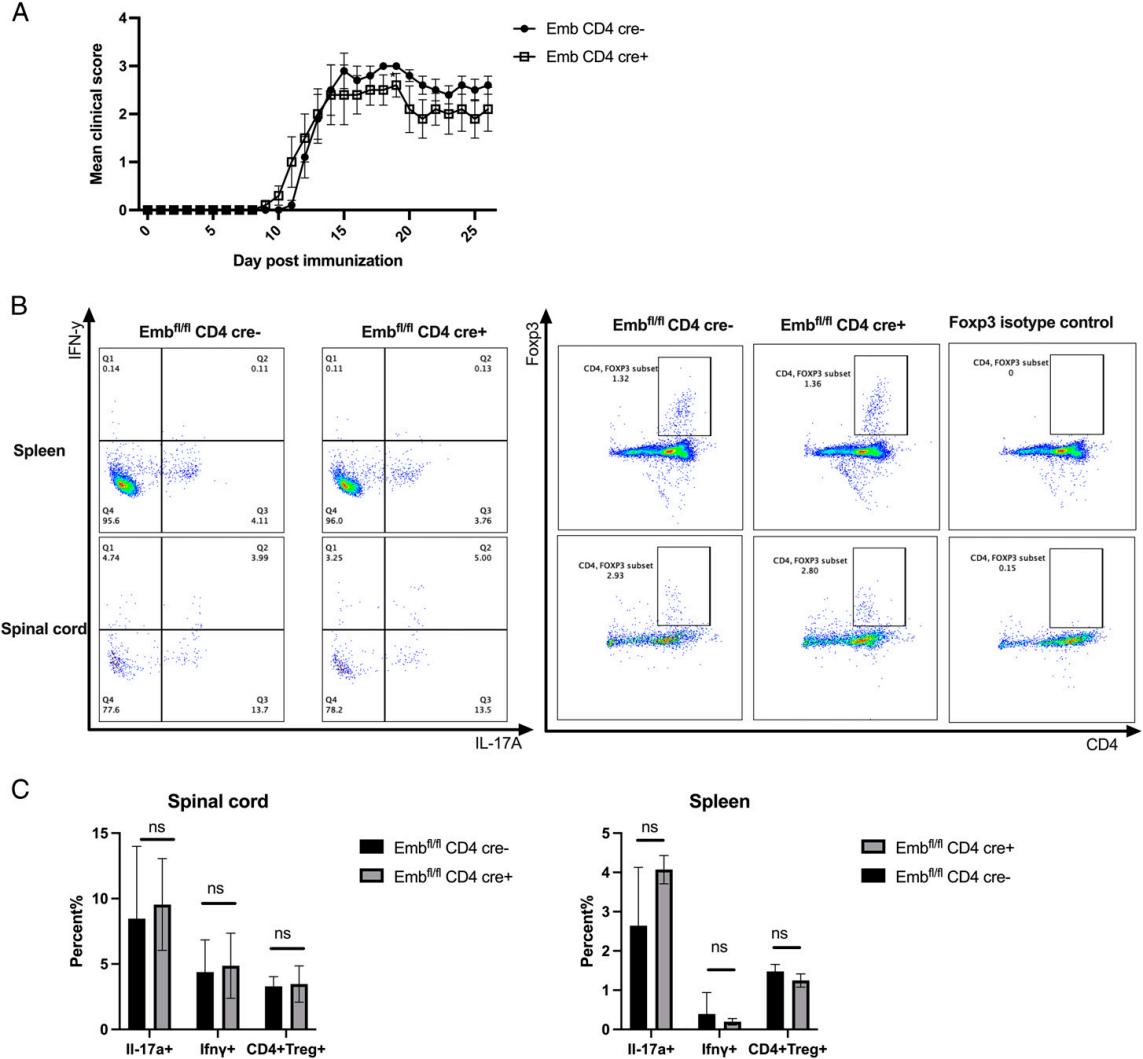
Embigin is dispensable for EAE pathogenesis. (**A**) WT and KO mice were immunized for EAE with MOG_35–55_ peptide, as described in *Materials and Methods*, and EAE score is shown (*n* = 5 per group, representative from two independent experiments). (**B**) Representative dot plots of spleen and spinal cord IL-17a^+^ cells, IFN-γ^+^ cells, and CD4^+^Foxp3^+^ cells in WT and KO mice. Mice were euthanized at day 30 after immunization. (**C**) Quantitative data of (B). Data are presented as means ± SEM. Significant differences were calculated with an unpaired Student *t* test.

To study the roles of *Emb* in the development of EAE, a disease model that requires Th17 cells, we immunized WT and KO mice with MOG_35–55_ peptide and monitored the disease by physical examination daily. There were no significant differences in clinical score (Fig. 4A). Also, there were no differences in the percent of IL-17A^+^ cells, IFN**-**γ^+^ cells, and Tregs between WT and KO mice, indicating that *Emb* is dispensable for EAE pathogenesis (Fig. 4B, 4C).

### T cell expression of *Emb* is dispensable for T cell priming and clearance of *Pneumocystis* infection

**FIGURE 5.**
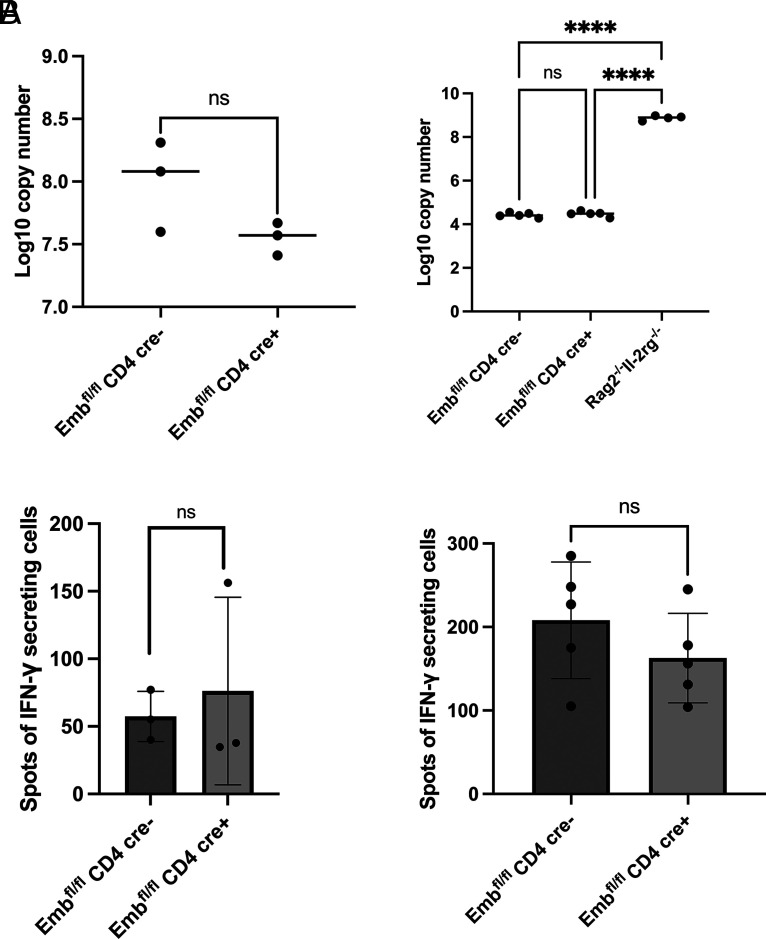
Embigin on CD4^+^ T cells is dispensable for T cell priming and clearance of *Pneumocystis* infection. (**A**) WT and KO mice were infected with 100 μl of *P. murina* inoculum (2 × 10^5^ cysts) via oral pharyngeal administration. Two weeks (left) (*n* = 3 per group from one experiment) or 6 wk (right) (*n* = 5 per group from one experiment) later, lungs from all mice were removed for RNA isolation and fungal burden assessment by RT-qPCR. (**B**) Two weeks (left) or 6 wk (right) later, single cells from mouse lungs were prepared for IFN-γ ELISPOT. Data are presented as means ± SEM. Significant differences were calculated with an unpaired Student *t* test or one-way ANOVA followed by a Tukey multiple comparison test. ****p ≤* *0.0001.

Clearance of *Pneumocystis* infection in mouse and humans requires CD4^+^ T cell immunity (27, 28). Thus, we infected WT and KO mice with *P. murina* by oropharyngeal aspiration. At 2 and 6 wk postinfection, the lungs were removed and RNA was extracted to assess fungal burden by RT-qPCR. No differences were detected between WT and KO mice (Fig. 5A). Also, part of the lungs was harvested to generate single-cell suspensions for IFN**-**γ ELISPOT under PC Ag stimulation. There were also no differences between WT and KO mice (Fig. 5B). These results suggest that *Emb* is dispensable for fungal CD4^+^ T cell priming and fungal clearance.

### *Emb* on CD8^+^ cells does not play a role in the influenza PR8 model

**FIGURE 6.**
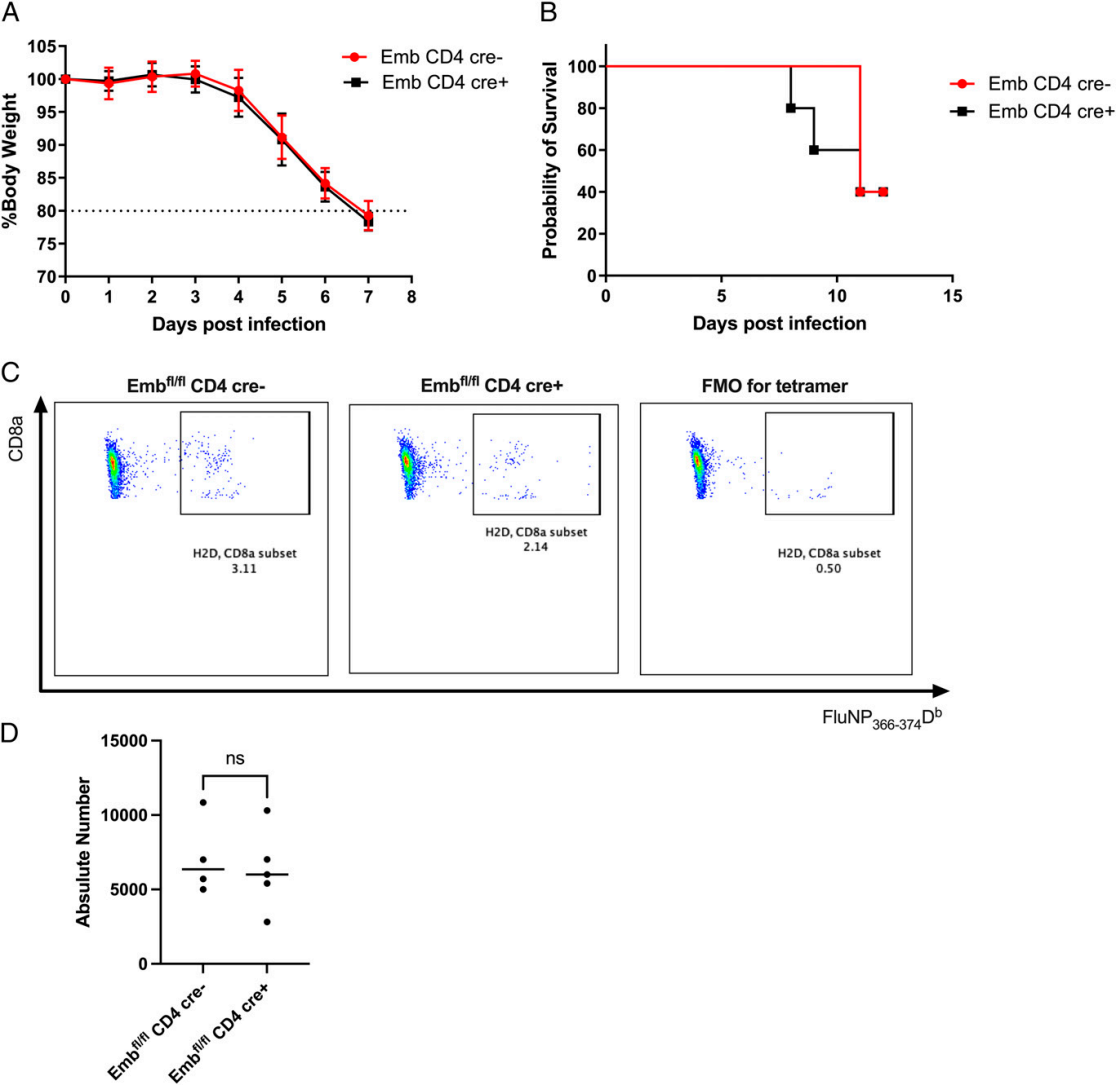
Embigin on CD4^+^ T cells does not play a role in the influenza PR8 model. WT and KO mice were infected by influenza H1N1 PR8 at a dose of 30 PFU. (**A**) Weight (*n* = 5 per group, representative from two independent experiments) and (**B**) survival were observed postinfection (*n* = 5 per group, representative from two independent experiments). Significant differences were calculated using the log-rank test. The *p* values were not significant by a log-rank test. (**C**) Representative dot plots of CD8^+^FluNP_366–374_D^b+^ cells in WT and KO mice (*n* = 4 per group from one experiment). (**D**) Absolute cell number of H1N1 PR8 tetramer-specific CD8^+^ cells at 60 d postinfection (*n* = 4 per group from one experiment). Significant differences were calculated with an unpaired Student *t* test.

*Emb* is also highly expressed on CD8^+^ cells. Therefore, we tested *Cd4cre* × *Emb^fl/fl^* mice in the influenza PR8 model, which is a CD8^+^ cell–dependent model. WT and KO mice were infected with Influenza H1N1 PR8 at a dose of 30 PFU. The body weight and survival of the mice were monitored daily over time. Both WT and KO mice lost weight to 80% at day 7 postinfection in a similar trend (Fig. 6A). The probability of survival of WT and KO mice was the same (Fig. 6B). In addition, we used influenza H1N1 PR8vtetramer to detect the number of Ag-specific CD8^+^ cells from the lung at 60 d postinfection. Still, no differences were found between WT and KO mice, which indicated that *Emb* may not play a role in the influenza PR8 model (Fig. 6C, 6D).

## Discussion

Embigin (encoded by *Emb*) is a type I transmembrane glycoprotein within the IgSF. It is known to play a role in embryonic endoderm development and HSPC regulation (3, 16). However, few studies have been conducted investigating the role of embigin in lymphocytes. This study is (to our knowledge) the first to investigate the role of embigin in T cell development. Our results indicate that embigin does not play a significant role in T cell development. Importantly, however, note that hematopoietic stem cells emerge in the yolk sac and later migrate to fetal liver in the mouse, where primitive hematopoiesis takes place. During this stage, the initial wave of lymphopoiesis occurs, giving rise to the first lymphoid precursors (29). T cell development takes place in the thymus. The spleen then acts as a secondary lymphoid organ, where T cells mature after having completed their development in the thymus and bone marrow, respectively (29). This current study looked at T cell development in both the spleen and thymus. Although the results of this study indicate that embigin does not play a significant role in T cell development, future studies should be directed at looking at *Emb* KO in mouse embryos to determine whether it plays a role in the earliest stages of lymphopoiesis.

Embigin has been shown to serve as a chaperone for MCT2, which is necessary for the transportation of nutrients into and out of the cell (9). Because both MCT2 and embigin expression levels are increased in lymphocytes, and effector T cells predominantly engage in aerobic glycolysis to meet their biosynthetic and metabolic demands, one could hypothesize that both MCT2 and embigin play an important role in maintaining T cell homeostasis by shunting lactate out of the cell (13, 30). Previous studies have demonstrated that inhibition of MCT1 impairs mouse T cell proliferation in vitro, although studies on MCT2 function on T cell function are limited (14).

Based on our previous work, we identified a vaccine composed of the A1 domain of heat-labile toxin from *E. coli* (LTA1) adjuvant and OmpX from the K2 strain of *K. pneumoniae*, which can elicit serotype-independent protection against the K1 strain by generating lung CD4^+^ TRM cells (19). By single-cell RNA sequencing, we found that the CD4^+^ TRM cells as well as CD4^+^ T cells from naive spleen highly expressed *Emb*. Additionally, CD8^+^ T cells also highly expressed *Emb*. However, the function of embigin in αβ T cells has not been described.

By using *Cd4cre* × *Emb^fl/fl^* mice, we generated mice with homozygous deletion of *Emb* in both CD4^+^ and CD8^+^ T cells and used this line to test the role of embigin in several T cell–dependent models. In our study, deletion of *Emb* did not affect generation of CD4^+^ and CD8^+^ effector or memory responses in various T cell–dependent models. Our previous studies on lung CD4^+^ TRM cells found that these CD4^+^ TRM cells expressed several unique adhesion molecules such as embigin, and preferentially homed to lung (19). However, this study demonstrated that *Emb* was dispensable for the homing property of lung-derived CD4^+^ Th17 TRM cells to the lung.

Our research also was (to our knowledge) the first to link embigin to *Tlr2* and *Il1α* signaling. Both *Tlr2* and *Il1α* were elevated in *Emb*-deficient CD4^+^ T cells, which indicated that the *Tlr2* signaling pathway might be influenced by *Emb* expression. Bulk RNA sequencing of CD4^+^ TRM cells from both WT and KO mice revealed that *Tlr2* was elevated in *Emb*-deficient CD4^+^ mice, suggesting that *Emb* might have an inhibitory effect on *Tlr2* signaling. CD4^+^ T cell–intrinsic *Tlr2* is known to help mediate Th17 development via the production of IL-6 and TGF-β (31, 32). Furthermore, *Tlr2* engagement with CD8^+^ T cells leads to increased activation, proliferation and memory cell development (33). This could suggest that embigin, through inhibition of *Tlr2*, decreases Th17 memory cell development; however, this was not reflected in the data in our experiments. The interplay between *Emb* and the *Tlr2* signaling pathway will require further study.

Additional research is required to elucidate the specific role of embigin within other organ systems such as the skin microenvironment. Interestingly, a recent study has shown that circulating conventional NK cells exhibit the ability to develop into long-lived tissue-resident NK cells in the mouse skin following acute infection, with embigin as a marker of interest in the transcriptional profile (20). Additional research is needed to explore the precise functions and interactions facilitated by embigin in T cells and NK cells within this context. These will provide valuable insights into the broader landscape of immune cell dynamics within tissues.

## Supplementary Material

Supplemental Figures 1 (PDF)
