# Supplementary material for: Embigin Is Highly Expressed on CD4+ and CD8+ T Cells but Is Dispensable for Several T Cell Effector Responses
Source: Immunohorizons. 2024 Mar 6;8(3):242–53. doi: 10.4049/immunohorizons.2300083 (PMC10985056; doi:10.4049/immunohorizons.2300083)
Supplement: Supplemental Figures 1 (PDF) [file IH_2300083_Supplemental_1.pdf]

Supplemental Figure 1

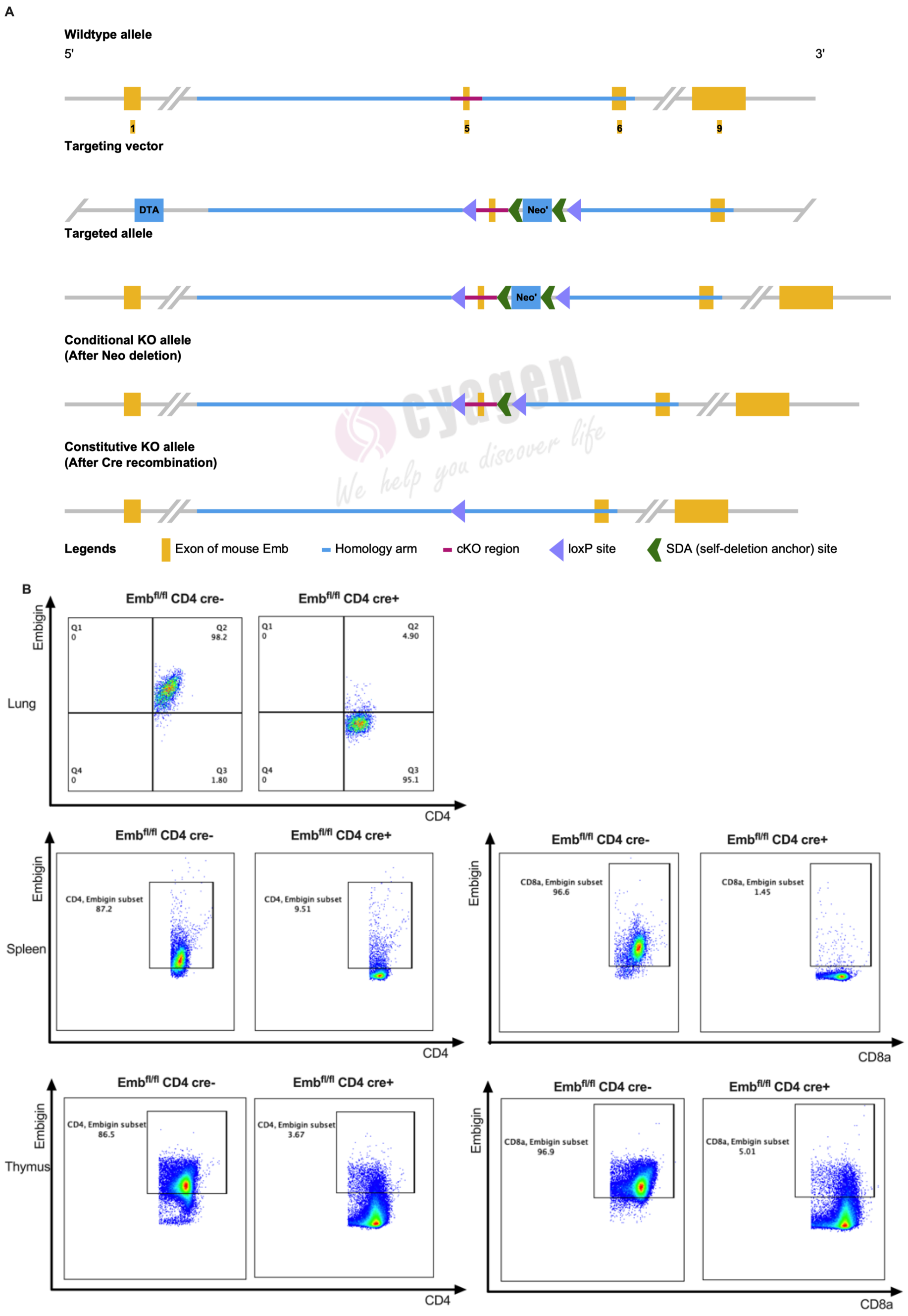

Figure S1. *Cd4cre* x *Emb*$^{fl/fl}$ mice generation and validation.
(A) Targeting strategy of *Cd4cre* x *Emb*$^{fl/fl}$ mice showing floxed exon 5. (B) Representative dot plots of Embigin expression on CD4$^+$ cells from lung CD4$^+$ TRM cells elicited by OmpX/LTA1 immunization, and CD4$^+$ and CD8$^+$ cells from spleen and thymus (n=3-5 from one experiment).

Supplemental Figure 2

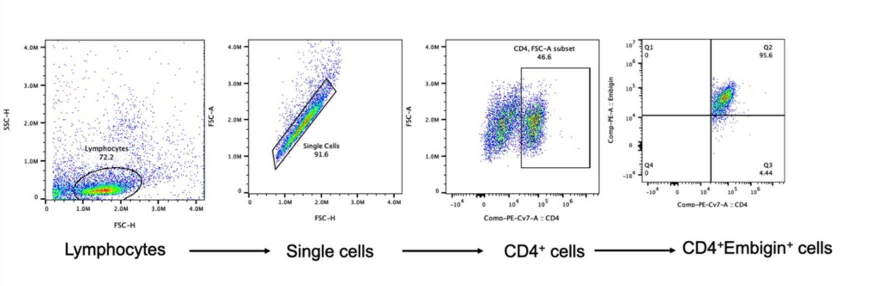

Figure S2. Gating strategy of Embigin on CD4+ cells.
OmpX/LTA1 immunized lung were used for flowcytometry. First, a size gate was applied to select for lymphocytes. Then the single cells were gated. $CD4^+$ were selected and $Embigin^+$ cells were gated from the $CD4^+$ cells.

Supplemental Figure 3

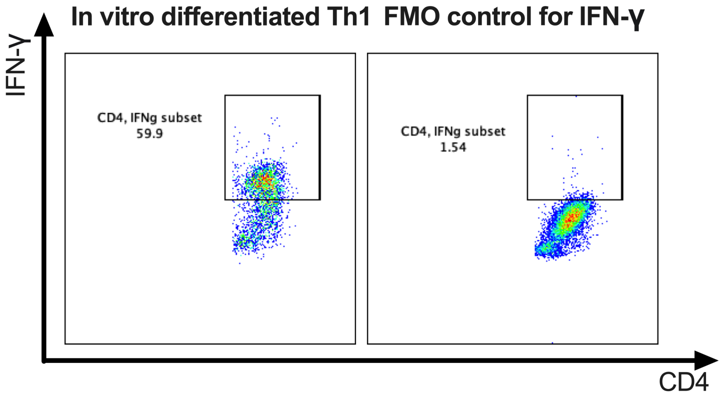

Figure S3. Intracellular staining validation for IFNγ.
Representative dot plots of IFN-γ expression on in vitro differentiated Th1 cells (left) and FMO control for IFN-γ staining (right). Naïve $CD4^+$ T cells were isolated from mouse spleen using Naive $CD4^+$ T Cell Isolation Kit (# 130-104-453, Miltenyi). The naïve $CD4^+$ T cells were differentiated to Th1 cells using CytoBox Th1 kit following manufacture's instruction (130-107-761, Miltenyi). Cells were collected for intracellular staining after 5 days differentiation (n=5, representative from two independent experiments).
